# New insights into mitophagy and stem cells

**DOI:** 10.1186/s13287-021-02520-5

**Published:** 2021-08-11

**Authors:** Qingyin Lin, Jiaqi Chen, Lifang Gu, Xingang Dan, Cheng Zhang, Yanzhou Yang

**Affiliations:** 1 Key Laboratory of Fertility Preservation and Maintenance, Ministry of Education, Key Laboratory of Reproduction and Genetics in Ningxia, Department of Histology and Embryology of School of Basic Medicine, Ningxia Medical University, Yinchuan, 75004 Ningxia People’s Republic of China; 2 The Agricultural College of Ningxia University, Yinchuan, 750021 Ningxia People’s Republic of China; 3 College of Life Science, Capital Normal University, Beijing, 100048 People’s Republic of China

**Keywords:** Mitochondria, Autophagy, Mitophagy, Stem cells, Cancer stem cells

## Abstract

Mitophagy is a specific autophagic phenomenon in which damaged or redundant mitochondria are selectively cleared by autophagic lysosomes. A decrease in mitophagy can accelerate the aging process. Mitophagy is related to health and longevity and is the key to protecting stem cells from metabolic stress damage. Mitophagy decreases the metabolic level of stem cells by clearing active mitochondria, so mitophagy is becoming increasingly necessary to maintain the regenerative capacity of old stem cells. Stem cell senescence is the core problem of tissue aging, and tissue aging occurs not only in stem cells but also in transport amplifying cell chambers and the stem cell environment. The loss of the autophagic ability of stem cells can cause the accumulation of mitochondria and the activation of the metabolic state as well as damage the self-renewal ability and regeneration potential of stem cells. However, the claim remains controversial. Mitophagy is an important survival strategy against nutrient deficiency and starvation, and mitochondrial function and integrity may affect the viability, proliferation and differentiation potential, and longevity of normal stem cells. Mitophagy can affect the health and longevity of the human body, so the number of studies in this field has increased, but the mechanism by which mitophagy participates in stem cell development is still not fully understood. This review describes the potential significance of mitophagy in stem cell developmental processes, such as self-renewal, differentiation and aging. Through this work, we discovered the role and mechanism of mitophagy in different types of stem cells, identified novel targets for killing cancer stem cells and curing cancer, and provided new insights for future research in this field.

**Supplementary Information:**

The online version contains supplementary material available at 10.1186/s13287-021-02520-5.

## Background

The lack of autophagy prevents cells from synthesizing proteins, such as lysosomal enzymes, respiratory chain proteins and antioxidant enzymes, leading to the accumulation of reactive oxygen species (ROS) [1] and reducing the content of mitochondrial DNA. Mitochondria, as semiautonomous organelles, can participate in various cellular functions, including ATP production, oxidative stress and calcium signal transduction [2]. If a cell undergoes a process such as proliferation or differentiation in the presence of inadequately functional mitochondria, it is likely to undergo a metabolic crisis [3], leading to cell death or senescence. Mitochondrial division and fusion are the mechanisms by which mitochondrial quality control can be evaluated. Mitochondria undergoing division are usually cleared from the cell through mitophagy, while the fusing mitochondria are protected from the effects of mitophagy [4].

Mitophagy is a specific autophagy phenomenon in which damaged or redundant mitochondria are selectively cleared by autophagic lysosomes. In the process of mitophagy, damaged or redundant mitochondria are “tagged” and surrounded by phagocytic vesicles that elongate to form a double-membranous vesicle of the autophagosome. The autophagosome fuses with the lysosome to form the autolysosome, releasing a set of potent lysosomal hydrolases to degrade enveloped mitochondria. Defected, unwanted, and aging mitochondria produce toxic byproducts, particularly reactive oxygen species (ROS), that threaten themselves, neighboring mitochondria, and host cells. As we age, toxic mitochondria continue to be produced and are removed through mitophagy. Mitophagy has been linked to aging, neurodegenerative diseases and cancer. Injuries to mitochondria release proapoptotic factors to induce apoptosis or self-elimination through autophagy, which helps to maintain cell viability [5]. Mitophagy can selectively clear dysfunctional mitochondria, but if defective mitochondria are not cleared in time, they become a source of oxidative stress and damage the health of the entire mitochondrial network. Studies have shown that decreased mitophagy speeds up the aging process, while enhanced autophagy maintains heart homeostasis and prolongs life [6]. Mitophagy may play a key role in delaying the accumulation of mitochondrial mutations in somatic cells [7]. Dysregulation of mitophagy is associated with the development of diseases and metabolic disorders. A variety of factors affect mitochondria, such as Atg32-mediated mitochondrial degradation through selective autophagy [8].

The role of mitophagy in several physiological and pathological processes has been summarized. However, the comprehensive role of mitophagy and its associated signaling pathways in stem cells have not been summarized.

Mitophagy also plays a key role in the maintenance and differentiation of stem cells, which include induced pluripotent stem cells (iPSCs), embryonic stem cells (ESCs), hematopoietic stem cells (HSCs), bone marrow mesenchymal stem cells (BMSCs), and cancer stem cells (CSCs). Stem cells are the mainstay of cell senescence. With age, stem cells lose their ability to divide, differentiate, and support tissue regeneration. Mitochondria regulate different metabolic and signaling pathways, but it has been reported that mitochondrial function decreases with stem cell aging. Stem cells provide material for the study of mitochondrial metabolism and the differentiation of cells into specific tissues [9–12]. Mitochondria play a direct role in maintaining the function of stem cells [13]. An increasing number of researchers have studied the energy- and metabolism-related characteristics of mitochondria in stem cells [12, 14]. Complete mitochondrial function is essential for stem cell differentiation, and mutations in mitochondrial DNA are also a major driver of stem cell aging. Therefore, as an important regulatory mechanism for mitochondrial homeostasis and function, mitophagy plays a vital role in stem cells.

## Mitophagy signaling pathway

**Fig. 1:**
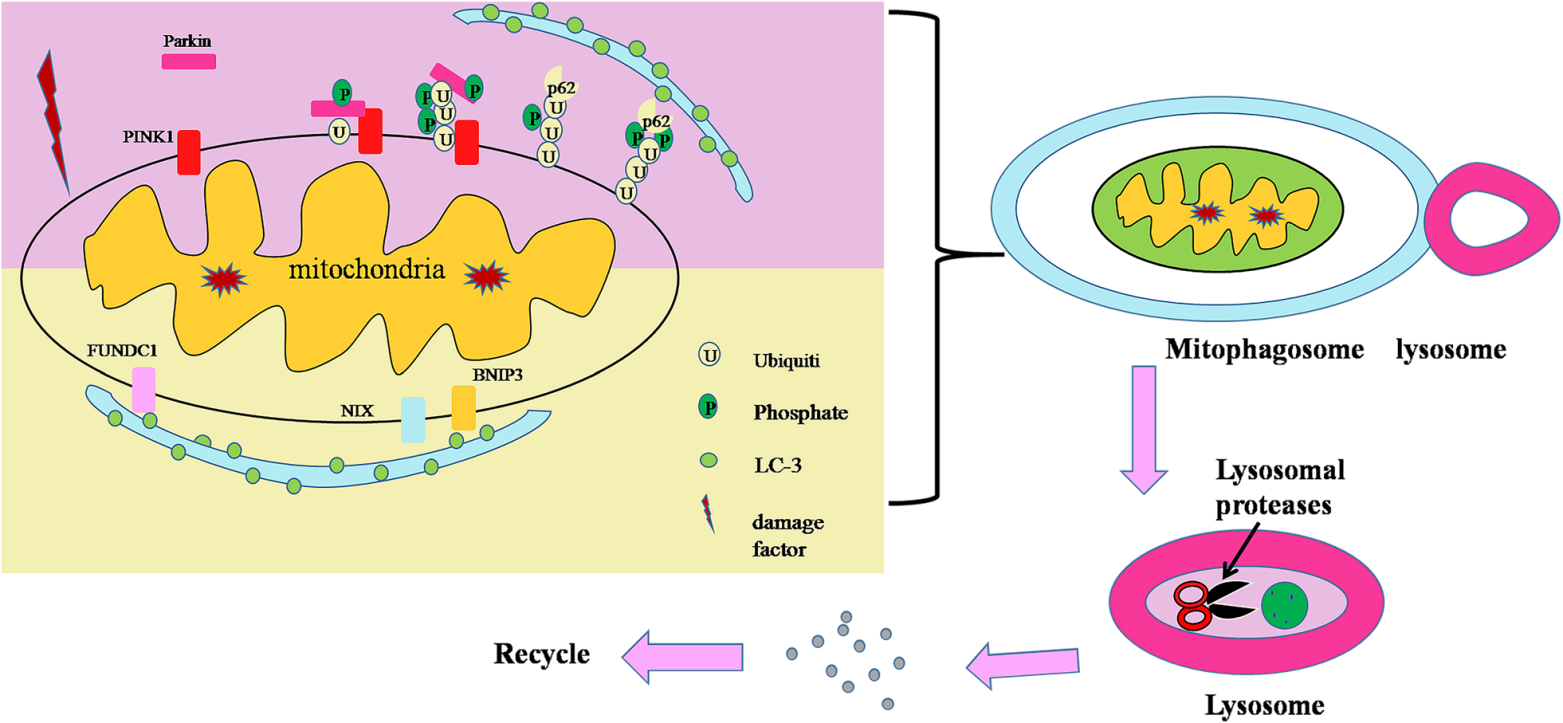
The pathway of mitophagy. PINK1 accumulates in large quantities after mitochondria are injured by the outside world. After Parkin is activated, autophagy receptor P62 is recruited, and autophagy receptors such as P62 bind with LC-3 to induce mitophagy. There are special structures on NIX, BNIP3 and Fundc1 that can directly bind to LC-3 to induce mitophagy. The damaged mitochondria are first swallowed by autophagosomes to form mitochondria, and then fuse with lysosomes, so that the defective mitochondria are degraded by lysosomal proteases. Finally, the degraded material is transported back to the cytoplasm for recycling

Mitophagy signaling pathways have been extensively studied. Currently, mitophagy signaling pathways mainly include the PINK1 (PTEN induced putative kinase 1)/Parkin (E3 ubiquitin ligases) signaling pathway, BNIP3 (BCL2/adenovirus E1B 19 kDa interacting protein 3-like) /NIX / (Nip3-like protein X) signaling pathway and FUNDC1 (FUN14 domain containing 1) signaling pathway (Fig. 1).

**Fig. 2:**
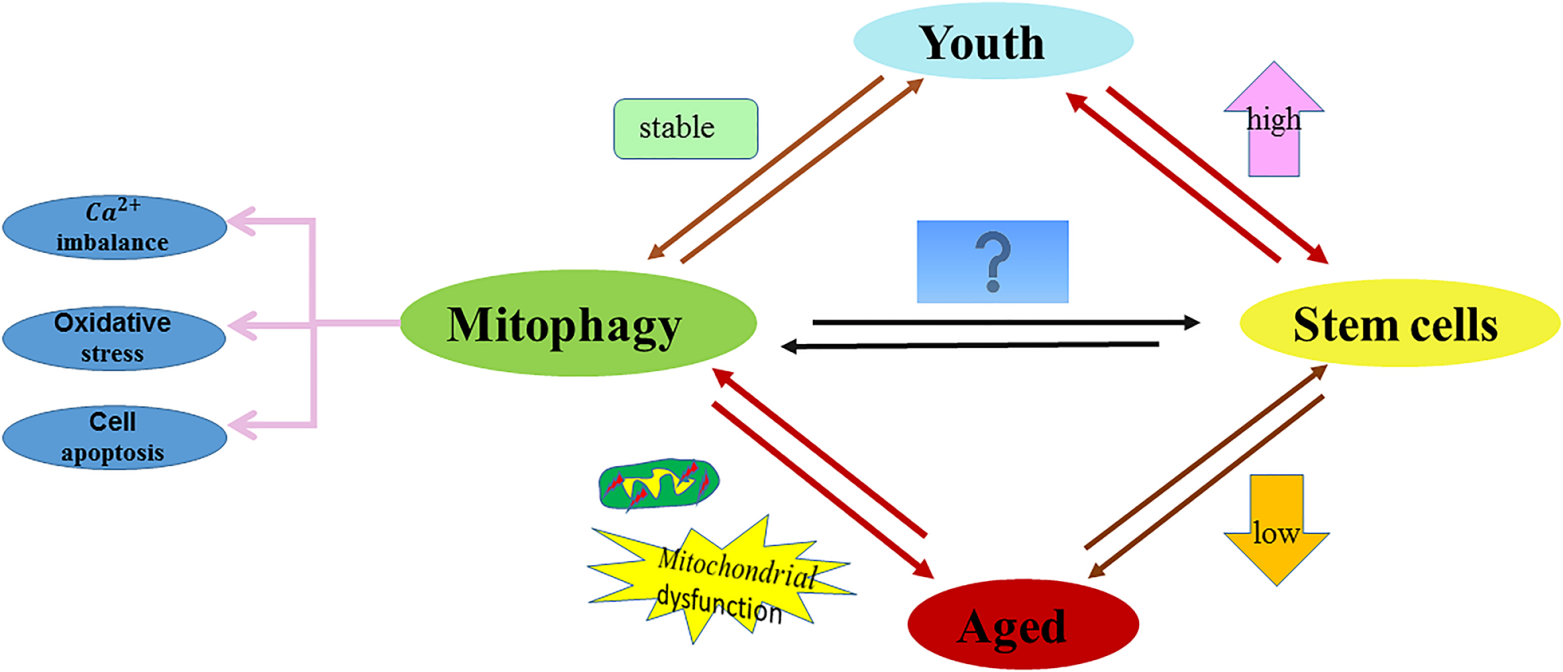
Relationship between mitophagy and stem cells. Mitophagy and stem cells change with age, but the specific relationship between mitochondrial autophagy and stem cells is still not clear

1. PINK1/Parkin-mediated mitophagy is a type of ubiquitin-dependent mitophagy [15]. PINK1 is necessary for the differentiation of mammary epithelial cells. It can promote the selective conversion of mitochondrial RC (respiratory chain) subunits and help maintain mitochondrial function and morphology. The activation of the PINK1/Parkin autophagy signal depends on the opening and closing of the TIM23 (translocase of the inner mitochondrial membrane 23) pore channel; when mitochondrial injury occurs, the TIM23 pore channel closes, blocking PARL's cleavage of PINK1, a key protein in autophagy, so that PINK1 accumulates on the outer membrane of mitochondria, and then, Parkin is selectively recruited into damaged mitochondria to promote mitophagy. Activated Parkin can ubiquitinate the anion potential channel protein VDAC1 (voltage-dependent anion channel 1) in damaged mitochondria, which is recognized by the signal adapter protein P62/SQSTM1 (sequestosome 1) and then connects with Atg8 family homologous proteins (LC3, etc.) on the phagocytic membrane surface to initiate mitophagy [16]. Autophagy in the PINK1/Parkin pathway is also affected by its upstream regulator, currently known as PRDX6 (peroxiredoxin 6). When mitochondria are damaged, ROS (reactive oxygen species) production increases. Excessive ROS leads to a large accumulation of PRDX6, which activates the downstream PINK1/Parkin pathway to clear damaged mitochondria. Activation of PINK1 protein kinase promotes mitophagy, and the PINK1/Parkin pathway is involved in various axes of mitochondrial quality control, including division and fusion, mitophagy, transport, and biogenesis. Inhibition of either PINK1 or Parkin gene expression impaired mitochondrial clearance and increased accumulation of damaged mitochondria. Therefore, the PINK1/Parkin pathway is an important sensor of mitochondrial dysfunction and a mediator of mitochondrial quality control.
2. BNIP3 and Nix are proapoptotic BH3 proteins located in the outer membrane of mitochondria that can directly bind with LC3 (microtubule associated protein 1 light chain 3) to induce mitochondria recruitment to autophagosomes for degradation and simultaneously remove excessive ROS. As a member of the Bcl-2 family, BNIP3L/ NIX is a class of proapoptotic proteins. It can stimulate cell proliferation, inhibit mitochondrial functional content, and enhance redox homeostasis. Most mammalian mature erythrocytes lack mitochondria, and mitophagy is mainly mediated by BNIP3L/NIX to remove mitochondria. Mitophagy mediated by BNIP3L plays an important role in the regulation of mitochondrial network formation, mitochondrial function and the activity of newly differentiated oligoendocytes. Nix-mediated mitophagy is an effective pathway of the oncogene KRAS (Kirsten Rat Sarcoma Viral Oncogene Homologue), and it also plays an important role in erythrocyte autophagy. BNIP3 combines with LC3 to induce mitophagy, which is a therapeutic target for muscle movement disorders and other autophagy-related diseases. Studies have shown that hypoxia-inducible factor-1 (HIF-1) regulates hypoxia-induced cell death factors BNIP3 and NIX in human tumors in a dependent manner, and BNIP3-mediated mitochondrial respiratory injury induces mitochondrial transformation through activation of mitophagy.
3. FUNDC1 is a complete mitochondrial outer membrane protein, which is a three-step transmembrane protein composed of 155 amino acids and has highly conserved properties in most mammals. FUNDC1 is a hypoxia-induced mitophagy receptor that mediates mitophagy in mammalian cells. The terminus of FUNDC1 has a domain (LIR) that can directly bind to LC3, and its dephosphorylation can enhance its interaction with LC3, thereby promoting selective mitochondrial autophagy. Therefore, regulation of FUNDC1 can activate or inhibit mitophagy. Mitophagy mediated by BNIP3L and FDDC1 regulates mitochondrial network formation during cardiac progenitor cell differentiation [12]. Studies have shown that endogenous FUNDC1 gene knockout can prevent hypoxia-induced mitophagy in mammalian cells [17]. Although the role of mitophagy in several biological processes was emphasized, the role of mitophagy in stem cells is not fully known, and its roles in stem cells are complicated and dependent on the type of stem cell (Fig. 2).

## Effects of mitophagy on the development of hematopoietic stem cells

Hematopoietic stem cells (HSCs) remain at rest in the bone marrow to maintain their ability to renew themselves but also divide as needed. Organelles, such as mitochondria, sustain accumulated damage during these cell divisions, and this damage may ultimately impair the cell’s ability to renew itself. HSCs in the bone marrow that produce blood cells, and research has found that mitochondria may be involved in this process. The fusion protein MfN1 (mitofusin 1/2) and fission protein FIS1 (mitochondrial fission protein 1) play a key role in erythropoiesis. Overexpression of FIS1 leads to the breakdown of the mitochondrial net, which inhibits both hemoglobin biosynthesis and erythroid differentiation, keeping cells in the immature differentiation stage [18]. Mitophagy inhibits the metabolism of HSCs, which is increasingly necessary to maintain the regenerative capacity of older hematopoietic stem cells. Hematopoietic defects in stem cells are associated with mitochondrial dysfunction of HSCs, whereas HSCs undergoing self-renewal division in vitro do not require mitochondrial activity [19]. Loss of Atad3a (ATPase family, AAA domain containing 3A) caused accumulation of PINK1 and activated mitophagy. Notably, deletion of PINK1 in Atad3a-deficient mice significantly rescued the mitophagy defect, resulting in restoration of the progenitor and HSC pools. Furthermore, Atad3a suppresses PINK1-dependent mitophagy and thereby plays a key role in hematopoietic homeostasis. The mitophagy factor PINK1 does not regulate the mitophagy of basal platelets but is essential for platelet function [20]. Asymmetrically isolated mitochondria provide the cellular memory of HSCs and drive MSCs to induce and detect autophagy in older HSCs [21]. Lysosomes can decompose biological macromolecules in vitro or on their own. Studies have shown that inhibition of lysosomal activity can keep HSCs static and potent, which may be related to the treatment of diseases. The role of Ca^2+^ in mitochondria has also been studied. After an increase in Ca^2+^ levels in mitotic HSCs, HSCs began to divide, indicating that Ca^2+^ can promote HSC mitosis [22]. Electron transport chain complex II maintains high mitochondrial membrane potential in HSCs. Although it has been reported that increased mitophagy leads to low mitochondrial quality in HSCs, the mitochondrial turnover capacity of HSCs is already low, so new studies on HSCs and mitophagy are needed [23].

Mitophagy maintains the functions of HSCs mainly through the mitochondrial metabolism process, and the role of mitophagy in HSCs is largely unknown.

## Effects of mitophagy on the development of germ stem cells

Reproductive stem cells are divided into female and male germ stem cells, but little is known about of mitophagy in male and female germ stem cells.

Spermatogonial stem cells rely heavily on the integrity of mitochondria to maintain their normal function, so mitochondrial defects can lead to premature senescence of spermatogonial stem cells. The ROS-induced effector small Maf in Drosophila testes maintains the differentiation of germ stem cells [24]. In addition, sperm mitochondrial function is critical for fertilization, and loss of sperm function is closely related to changes in mitochondrial integrity, including defects in mitochondrial ultrastructure, mitochondrial genome, and transcriptome, as well as low mitochondrial membrane potential or increased ROS [25, 26]. Additionally, the importance of autophagy in sperm was emphasized by several studies [27–29].

Although the role of mitophagy in female and male stem cells has rarely been reported, the importance of mitochondria and autophagy in female and male germ cells provides some evidence that mitophagy might play important roles in female and male germ stem cells and will be explored in future studies.

## Effects of mitophagy on the development of iPSCs/ESCs

Induced pluripotent stem cells (iPSCs) have strong differentiation potential. They have fewer mitochondria than somatic cells and are immature and mainly rely on glycolysis as an energy source [30]. Mitochondria play an important role in the regulation of iPSCs [31]. Mitochondrial integrity is an important prerequisite for the self-renewal and differentiation of iPSCs. The accumulation of damaged mitochondria is a hallmark of aging and age-related neurodegeneration, including Alzheimer’s disease (AD). The molecular mechanisms of impaired mitochondrial homeostasis in AD are currently being investigated. Here, we provide evidence that PINK1/Parkin-mediated mitophagy is impaired in the hippocampus of AD patients, in induced pluripotent stem cell-derived human AD neurons, and in animal AD models, that impaired removal of defective mitochondria is a pivotal event in AD pathogenesis and that mitophagy represents a potential therapeutic intervention [32].

Mitophagy-driven mitochondrial regeneration may contribute to the ability of iPSCs to inhibit differentiation by guiding biological energy conversion and metabolic remodeling properties. The PINK1-dependent mitophagy pathway is an important mitochondrial switch that determines the efficiency and quality of somatic reprogramming, and loss of PINK1 reduces the speed and efficiency of iPSC reprogramming. In addition, the AKT (protein kinase B) pathway can also regulate PINK1-dependent mitophagy in iPSCs. Mitophagy is impaired in neurons derived from iPSCs cells in AD. Interestingly, the accumulation of ROS and susceptibility phenotypes of Park2-iPSC-derived AD neurons can be detected [33].

In contrast to differentiated cells, human embryonic stem cells (ESCs) have mitochondrial characteristics and mechanisms that are prone to apoptosis. It is not clear whether ESCs are susceptible to autophagy, but there are some related studies. PHB2 (prohibitin 2) regulates embryonic cell lineage specific-differentiation in mitochondria [34]. PHB2 is an inner mitochondrial membrane mitophagy receptor [35]. Cardiac myocytes derived from hESCs with BMAL1 deficiency exhibit typical phenotypes of dilated cardiomyopathy, including reduced contractility, maladjustment of calcium, and myofilament disorders, as well as compromised mitophagy and mitochondrial dysfunction through reduced BNIP3 protein levels [36]. Kaempferol is a natural flavonoid found in a normal diet. At high concentrations, kaempferol is harmful to mouse ESCs and increases the production of mitochondrial ROS [37].

Hence, mitophagy is involved in the self-renewal and differentiation of iPSCs and ESCs, and the dysfunction of mitophagy contributes to the production of mitochondrial ROS.

## Effects of mitophagy on mesenchymal stem cells

Mesenchymal stem cells (MSCs) were firstly indentified in bone marrow, and nowadays MSCs can be isolated from bone marrow, fat, synovial membrane, bone, muscle, lung, liver, pancreas and other tissues as well as from amniotic fluid and umbilical cord blood, however, the most used MSCs are those of bone marrow origin. MSCs have immunomodulatory actions and exert mitoprotective effects that attenuate the production of ROS and promote the restoration of tissue function and metabolism after perinatal insults [38].

MSCs protect tissues from cell death due to ischemia/reperfusion injury [39]. The synergistic regulation of mitochondrial biogenesis and antioxidant enzymes in the osteogenic differentiation of MSCs is synergistic [40]. However, mitochondria play an important role in this process; that is, somatic mitochondria alert MSCs to dangerous conditions and then promote an adaptive repair response [41].

Environmental stress during Bone marrow mesenchymal stem cells (BMSCs) transplantation or in damaged tissues can lead to catastrophic problems, such as cytotoxicity and poor survival of BMSCs. Mitophagy plays an important role in promoting mitochondrial division of BMSCs [3]. Mitophagy promotes the stemness of bone marrow-derived mesenchymal stem cells by regulating mitochondrial fission, and inhibition of mitophagy suppresses the stemness of BMSCs [42]. BMSCs protect leukemia cells from the effects of chemotherapy, but the mechanism is unclear.

In aging MSCs, replicative aging leads to the aggravation of mitochondrial dysfunction by inhibiting mitophagy. Melatonin can protect MSCs from the effects of replication aging during the in vitro amplification process by mitochondrial quality control [43]. Melatonin increases the expression of HSPA1L (heat shock 70 kDa protein 1L), thereby upregulating mitophagy and prolonging the survival time of cells under oxidative stress conditions [44]. Therefore, melatonin can protect BMSCs from the effects of replicative aging during in vitro amplification through mitochondrial quality control [43]. Other studies have shown that BMSCs aged more severely in patients with idiopathic pulmonary fibrosis [45].

MSCs upregulated PINK1-dependent mitophagy to participate in liver protection [46]. BMSC injection restored PINK1/Parkin-mediated mitophagy, improved mitochondrial dysfunction and reduced endothelial cell apoptosis in diabetic rats. Therefore, MSCs may improve mitochondrial dysfunction through PINK1/Parkin-mediated mitophagy, thereby protecting endothelial cells from damage caused by hyperglycemia [47, 48] The upregulation of Parkin expression and downregulation of p53 expression in BMSCs can significantly enhance the mitophagy ability of BMSCs and reduce the accumulation of damaged mitochondria in cells, effectively resisting stress-induced apoptosis and senescence of BMSCs and improving the effect of BMSCs transplantation on hormone-induced early ONFH (osteonecrosis of the femoral head) [48]. Induction of mitophagy plays an important role in protecting BMSCs from oxidative stress [49]. PD (Placenta-Derived)-MSCs regulate mitophagy factors in invasive trophoblasts by regulating the balance between PTEN-induced putative kinase 1 (PINK1) and Parkin RBR E3 ubiquitin protein ligase (PARKIN) expression [50].

Mitophagy plays a crucial role in maintaining cellular homeostasis and resisting oxidative stress, as this process can control the quality and quantity of mitochondria by eliminating dysfunctional or damaged mitochondria that lead to cell death. Notably, BMSC-mediated mitochondrial transfer can optimize cell function [51]. Cell adhesion-mediated mitochondrial transfer and studies on the drug resistance of leukemia cells induced by MSCs are expected to provide a new target for clinical treatment [52]. Simultaneously, studies have shown that MSCs have a protective effect on mitochondrial dysfunction induced by cigarette smoke in mice [53]. MF (Mangiferin) treatment regulates mitochondrial fission plasticity, increases mitochondrial DNA, and improves mitochondrial dynamic balance by inhibiting PINK1-PRKN-mediated mitosis. Studies have shown that MF promotes brown adipose cell phenotypes by inhibiting mitophagy in C3H10T1/2 MSCs [54].

Study shows that ADMSCs (adipose-derived mesenchymal stem cells) differentiate into pancreatic cancer-associated fibroblasts in vitro [55]. ADMSCs protect the lung from ischemia reperfusion (IR) injury by attenuating inflammation/oxidative stress/mitochondrial damage/autophagy signaling pathways [56]. ADMSCs transplantation can effectively repair rat ovarian damage caused by CTX, and its mechanism may be related to the inhibition of mitochondrial apoptosis of granulosa cells [57]. However, there are few studies on the relationship between ADMSCs and mitophagy.

Therefore, the stemness of MSCs is maintained and controlled by mitophagy, and the dysregulation of mitophagy in MSCs causes apoptosis and senescence of MSCs. In addition, whether the role of mitophagy in MSCs is dependent on the different MSC types is unknown and needs to be explored.

## Effects of mitophagy on the development of neural stem cells

Neural stem cells (NSCs) are self-renewing pluripotent stem cells located in different niches in the subventricular area of the lateral ventricle and the subgranular area of the hippocampal dentate gyrus in adults. The descendants of NSCs, called neural precursor cells, can proliferate and differentiate into the three main cell types of the nervous system: neurons, astrocytes, and oligodendrocytes. Reduced autophagy pathways in neurons lead to human neurodegenerative disease, which can be promoted by iPSC NSCs in Alzheimer's disease [58]. There are few studies on the role of neural stem cells in mitophagy, but there are some studies on the dynamics of mitochondria. Mitophagy was inhibited by miR-137, and miR-137 regulates mitochondrial dynamics by inducing mitochondrial fusion and division, leading to increased mitochondrial content and regulating the fate of NSCs [59]. Thyroid hormone signals trigger specific metabolic changes, involving changes in mitochondrial activity and mitochondrial dynamics, and regulate the fate of NSCs from development to aging [60]. Indeed, mitophagy was regulated by thyroid hormone signals in several studies [61–64].

Stem cells from human exfoliated deciduous teeth (SHEDs) are a kind of stem cell that can differentiate into nerve cells, and mitochondrial activity is involved in the neuronal differentiation of stem cells [65].

Therefore, mitophagy is involved in the development of NSCs, but the actual role of mitophagy in NSCs has not been fully elucidated. Whether mitophagy is involved in differentiation, self-renewal, and aging needs to be explored.

## Effects of mitophagy on cancer stem cells

Cancer stem cells (CSCs), also known as tumor initiating cells, are highly tumorigenic subsets of cells within a tumor. They have the dual characteristics of cancer cells and stem cells, including self-renewal, multidirectional differentiation potential and chemotherapy resistance. Many CSC phenotypes, including metabolic and cell signaling pathway activity, are due to changes in mitochondrial function and turnover, which are regulated by continuous mitochondrial fusion and division cycles [66]. CSCs’ mitochondria are ancient organelles at the crossroads of new anticancer therapies. Mitochondria play a crucial role in the regulation of stem cell recognition, differentiation and fate. A similar role is found in CSCs, which have been implicated in the progression and drug resistance of many tumors. The acquisition and maintenance of mitochondrial and CSC phenotypes were summarized in the literature [67]. Mitochondria can regulate cell metabolism and avoid the elimination of apoptosis in cancer cells. Thus, the entire mitochondrial cycle, from biogenesis to death, either through mitophagy or through apoptosis, can be targeted by different drugs to reduce mitochondrial fitness and thus restore or increase sensitivity to chemotherapeutic drugs [68]. Mitochondrial biogenesis has become an important target for the study of anti-CSC drug resistance mechanisms due to the high mitochondrial content in CSCs [3]. The use of mitochondrial targeting therapy to target CSCs may be valuable, but there are challenges to mitochondrial targeting therapy [69]. Therefore, the method of regulating mitochondrial function in clinical practice may be a good anti-CSC drug. In addition, the role of chloroquine analogs combined with antibiotics in the autophagy process of CSCs has also been reported in the literature, which may provide the possibility for clinical anticancer strategies [70]. Mitochondrial dysfunction underlies many pathologies, including cancer. The effects of antibiotics on mitochondria in tumor and non-tumor cells are manifested by reduced mitochondrial membrane potential, reduced ATP production, morphologic changes, and reduced respiration, all of which are indicative of mitochondrial dysfunction (MDF). This is paralleled by increased levels of ROS and reduced activity of the mitochondrial respiratory complex. However, the survival and reproduction of cancer cells were not significantly affected by antibiotics, which may be due to glycolytic transformation or activation of autophagy. Inhibition of autophagy and antibiotic therapy significantly reduced the tumorigenicity of cancer cells, suggesting a potential strategy for anticancer therapy [71].

CSCs utilize mitophagy as the main survival response mechanism for their growth, reproduction and tumorigenicity. Mitochondrial biogenesis is an important cellular event that replaces damaged mitochondria through the synergistic regulation of multiple transcription factors to meet the biological energy requirements of cells. Mitophagy and its influence on the regulation of CSC behavior during tumorigenesis have been reported in the literature [72]. The interesting aspects of mitochondrial reconnection involved in mitophagy and mitochondrial biogenesis in CSCs have been summarized in the literature [73]. The desiccation and differentiation of CSCs were also discussed. Autophagy often helps cancer cells survive during long periods of dormancy and the final growth of metastatic disease. An increasing number of studies are attributing the stemness of cells to mitophagy [74]. Recently, an enzyme called sirtuin, which is similar to mitochondrial autophagy proteins, was discovered. Sirtuins are NAD^+^-dependent deacetylases that have been implicated in aging, oxidative stress control, inflammation, differentiation, and cancer. How sirtuins control mitophagy in cancer cells has been discussed in the literature [3].

PINK1-mediated mitophagy controls the activities of p53 to maintain hepatic CSCs [75]. PINK1 plays an important role in tumorigenesis, progression and recurrence. Mitochondria also play a role in liver cancer stem cells (LCSCs). In LCSCs and HBx-expressing HCC cells, BNIP3L-dependent mitophagy was also activated, thus triggering a metabolic transition to glycolysis. LCSCs have a high level of BNIP3L dependence. Mitochondrial and energy metabolism-related characteristics can be used as a new indicator of LCSCs [76]. The essential role of mitophagy in hepatic cancer stem cells was summarized by Lee et al. [77], and mitophagy is involved in the differentiation and apoptosis of hepatic cancer stem cells.

Inhibition of the mitogen-activated protein kinase (BMK1) pathway inhibits cancer stem cells through BNIP3 and BNIP3L, and BMK1 plays an important role in maintaining the stemness of CSCs. Hypoxia-induced autophagy is mediated by the BH3 domain, and autophagy via BNIP3L is a survival mechanism that promotes tumors. BNIP3L, located at 8p21, isfound in ovarian cancer heterozygous loss (LOH), breast cancer heterozygous loss (LOH) and prostate cancer metastatic suppression regions, has homology with pro-apoptotic protein sequences, and can inhibit soft agar colonization. Interestingly, studies have reported that BNIP3L is unlikely to be a target for ovarian or breast 8p-LOH [78], but BNIP3 may contribute to pancreatic cancer resistance to hypoxia-induced cell death.

Mitochondrial damage agents can be used to prevent the maintenance and proliferation of CSCs to better control tumor disease. Mitochondrial biosynthesis is crucial to the expansion and survival of CSCs, so mitochondria have become an important target for new therapeutic pathways. Glioblastoma is a type of invasive malignant tumor due to the presence glioblastoma tumor stem cells, so separation of MSC mitochondria can be transferred to glioblastoma cells. Further analysis of mitochondria transfer cells can help determine the role of exogenous mitochondria in cell metabolism and plasticity and the effects on biological characteristics, such as proliferation and response to treatment. In addition, ovarian cancer stem cells have a unique mitochondrial phenotype, and Bcl-Xl (Bcl-2 family proteins) is a key regulator of their stability. Mitochondria-related macrophage stimulating factor signaling proteins can be used totreat ovarian cancer resistance disease.

Overall, exploring the role and mechanism of mitophagy in CSCs, including differentiation, self-renewal and death, might be a novel target for treating cancer.

## Others stem cell and mitophagy

Leukemia stem cells (LSCs) are thought to drive the genesis of acute myeloid leukemia (AML) as well as relapse following chemotherapy, and AMPK (AMP-activated protein kinase)/FIS1 (Fission 1)-mediated mitophagy is required for self-renewal of human AML stem cells; therefore, inhibition of mitophagy might be a potential target for killing AML and LSCs [43].

The intestinal epithelium is the most active adult tissue in mammals, and the presence of intestinal stem cells (ISCs) supports this renewal process. We identified a protective pathway for ISCs that is mediated by a microorganism derived from mouse acyldipeptide (MDP) and revealed a link between mitosin-mediated ROS clearance and MDP stimulation by ISC bacteria. The specific mechanism is as follows: under stress conditions, the cytoplasmic sensor NOD 2 (binding oligomerization domain-containing protein 2) recognizes MDP, and its high expression level in ISCs mediates the lethal removal of ROS molecule overdose. The synergistic effect of autophagy proteins Atg16L1 (autophagy related protein 16 like protein 1) and NOD 2 mediates the activation of the mitophagy process, thereby eliminating damaged mitochondria and thus protecting ISCs. MDP induces a significant reduction in total ROS and mitochondrial ROS in intestinal stem cells, which is related to the induction of mitophagy. The mechanisms of NOD2-mediated cell protection include the synergistic activation of NOD2 and ATG16L1 through a nondependent nuclear factor κB (NF-κB) pathway and the elimination of lethal excess ROS molecules through mitophagy.

Adipose tissue-derived mesenchymal stem cells (ASCs) are pluripotent cells capable of differentiating into osteogenic, cartilaginous, and adipose lineages. ASCs have attracted wide attention in clinical applications due to their convenient and minimally invasive features. One study suggested that triggering selective mitophagy may be a rescue mechanism that allows ASCs to maintain pluripotence and survive in an adverse, proinflammatory adipose tissue microenvironment. Therefore, targeted mitophagy in ASCs may become a new therapeutic approach in the future, aimed at improving mitochondrial biogenesis and metabolism, thereby influencing cell fate.

Amniotic fluid stem cell (AFSC) transplantation is a promising therapeutic strategy for the treatment of diabetic nephropathy, and sirtuin-3 (SIRT3) is a new mitochondrial protective factor. SIRT3 expression was positively correlated with the survival and proliferation of AFSCs. Mitophagy that restores SIRT3 activation can protect AFSCs from high glucose-induced apoptosis by protecting mitochondrial function, so the overexpression of SIRT3 in AFSCs may further improve the efficiency of stem cell therapy.

## Conclusion and perspectives

**Fig. 3:**
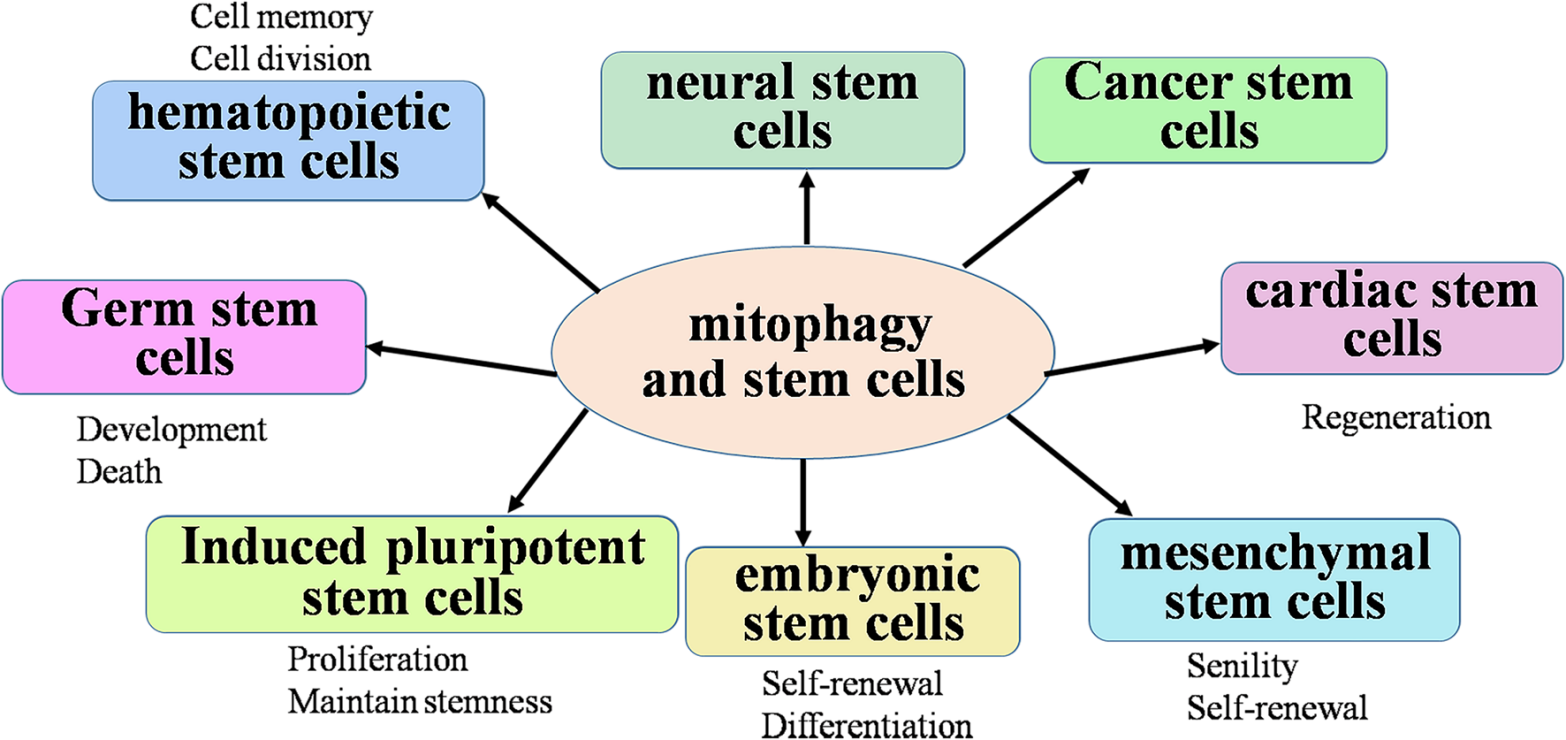
The roles of mitophagy in stem cells. Mitophagy not only regulates bioenergetics, cell death, calcium dynamics and reactive oxygen species (ROS) generation but also actively participates in many aspects of stem cell function, including self-renewal and differentiation proliferation, survival and apoptosis, to maintain stem cell multipotency

Mitochondria play an essential role in stem cells and mediate stem cell fate and aging; therefore, stem cell homeostasis is controlled by mitochondria. The stemness and fate of stem cells are damaged by mitochondrial dysfunction. Furthermore, mitochondrial homeostasis is regulated by mitophagy, which might emphasize the vital role of mitophagy in stem cells. Mitophagy and most regulators of mitophagy play important roles in hematopoietic stem cells, germ stem cells, iPSCs/ESCs, mesenchymal stem cells, neural stem cells, cancer stem cells and others (Fig. 3, Table 1; references in Additional file 1). However, the role and accurate pathway of mitophagy in stem cells are complicated and might be dependent on stem cell type.

**Table 1:** List of modulator by mitophagy in stem cell or mitochondria modulator in stem cell

Modulator	Functions	References
GP78	The increase of Gp78/AMFR (Gp78/autocrine motility factor receptor) expression and AMF (autocrine motility factor) internalization level in PTC (papillary thyroid carcinoma) is related to the expression of cancer stem cell markers	[1]
HIF-1 (hypoxia-inducible factor 1)	HIF1 mediates nuclear localization and TAZ (transcriptional co-activator with PDZ-binding motif) expression to induce the breast cancer stem cell phenotype HIF1A reduces compression-induced apoptosis of nucleus pulposus stem cells by up-regulating autophagy	[2] [3]
TBK1 (TANK binding kinase 1)	Zika virus disrupted the localization of phosphorylated TBK1 and mitosis in human neuroepithelial neural stem cells and radial glia Retinoic acid aggravates ATG10 (Autophagy Related 10)-dependent autophagy damage in TBK1 mutant hiPSC s -derived motor neurons through SQSTM1/p62 accumulation The replication of TBK1 stimulates autophagy in iPSC -derived retinal cells Knockdown of TBK1 decreases Pca (prostate cancer) stem-like cells drug resistance in vivo and in vitro	[4] [5] [6] [7]
OPTN (optineurin)	OPTN protects ESC mitochondrial homeostasis and pluripotency by eliminating damaged mitochondria through TBK1-activated OPTN binding of PINK1 -phosphorylated Ubiquitin OPTN regulates bone fat balance and the fate of mesenchymal stem cells during aging by clearing FABP3 (fatty acid binding protein 3)	[8] [9]
NIX(Nip-like protein X)	Silencing NIX impaired cancer stem cell maintenance, mitochondrial reactive oxygen species clearance	[10]
BNIP3 (Bcl2/adenovirus E1B 19 kDa protein-interacting protein 3)	Hypoxia induces BNIP3 to stimulate the production of FASN (fatty acid synthase)-dependent free fatty acids and enhance the therapeutic potential of human mesenchymal stem cells derived from cord blood Angelica polysaccharide down-regulates BNIP3 to regulate autophagy and apoptosis induced by hypoxia in rat neural stem cells Promoting the expression of miR-210-3p can prevent NSC from hypoxic injury, which may reduce NSC cell apoptosis and AIF and BNIP3 expression levels MiR-24-3p down-regulates BNIP3 in GSC to inhibit mitophagy	[11] [12] [13] [14]
BMAL-1(Brain and muscle Arnt-like protein-1)	BMAL1 deficiency in hESC cardiomyocytes reduces BNIP3 protein levels and leads to impaired mitophagy	[15]
Pink1(PTEN-induced kinase 1)/Parkin	Intestinal stem cell (ISC)/EB-specific knockdown PINK1 or Parkin suppresses the age-related loss of tissue homeostasis Parkin mediates mitophagy in insulin-deprived HCN (hippocampal neural stem) cells Parkin-mediated mitophagy is necessary for the differentiation of MuSCs (Muscle stem cells) and plays a key role in skeletal muscle regeneration In induced pluripotent stem cells, the endogenous level of Parkin is insufficient to initiate mitophagy after the loss of mitochondrial membrane potential	[16] [17] [18] [19]
OPA1(optic Atrophy 1)/MFN (mitofusin)	Melatonin exerts a protective effect on Cr (VI)-induced mitophagy by restoring METTL3 (methyltransferase like 3)-mediated RNA mA modification and activating mitochondrial fusion proteins MFN2 (mitofusin2) and OPA1 (Optic Atrophy 1)	[20]
Miro (mitochondrial Rho GTPases)	Miro fixes mitochondria on the microtubule motor and is removed as an early step to clear dysfunctional mitochondria to prevent mitochondrial movement Miro1 overexpression leads to increased stem cell repair	[21] [22]
Fis1(mitochondrial fission factor)	AMPK (AMP-activated protein kinase)/FIS1 mediated mitophagy contributes to the self-renewal of human AML (acute myeloid leukemia) stem cells FIS1 promotes the stemness of human lung cancer stem cells through mitophagy	[23] [24]
Drp1 (Dynamin-related protein 1)	Drp1 is required for differentiation of embryonic stem cells	[25]
ULK1 (Unc-51-like kinase 1)	The phosphorylation of ULK1 by AMPK (AMP-activated protein kinase) is essential for the stemness regulation of ESC The p53 activity in mouse embryonic stem cells is not required for the upregulation of ULK1-dependent autophagy	[26] [27]
PCK2 (mitochondrial phosphoenolpyruvate carboxykinase)	PCK2 regulates the osteogenic differentiation of MSCs through autophagy-activated kinase 1 (ULK1)-dependent autophagy	[28]
PHB2 (Prohibitin 2)	PHB2 is a key mitochondrial regulator for homeostasis of embryonic stem cells	[29]
Apelin-13	Apelin-13 induces mitophagy and improves oxidative stressin bone marrow mesenchymal stem cells	[30]
MAPK (mitogen-activated protein kinase)	Bone marrow mesenchymal stem cells repair Cr (VI) damaged kidneys through mitophagy mediated by MAPK signaling pathway	[31]
KLF2 (Kruppel-like factor 2)	KLF2 regulates the differentiation of dental pulp stem cells by inducing mitophagy	[32]
METTL3 (methyltransferase like 3)	Melatonin protects mitophagy by restoring METTL3-mediated RNA mA modification	[20]
PEDF (Pigment epithelium-derived factor)	PEDF in placenta-derived mesenchymal stem cells (PD-MSCs) facilitate mitophagy and restore the loss of visual cycles in HO-injured rat retinas	[33]
Bhlhe40/Sirt1 (Sirtuin-1)	Bhlhe40/Sirt1 axis regulated mitophagy in neural stem cells Sirt1selectively clears EGFR-TKI resistant CSCs by regulating mitochondrial oxidative phosphorylation in lung adenocarcinoma cells	[34] [35]
SQSTM1/p62 (sequestosome 1)	SQSTM 1 has an effect on the early dependence of mitophagy, and its loss will lead to changes in the mitochondrial gene expression and function of iPSC-derived neurons	[36]
LRRc17 (leucine-rich repeat containing 17)	Knockout of LRRC 17 gene can rejuvenate aging bone marrow mesenchymal stem cells (BMSC)	[37]
Sirt3 (Sirtuin-3)	Sirt3-mediated mitophagy regulates AGEs (advanced glycation end products)-induced senescence of BMSCs SIRT3 protects mitochondrial homeostasis by regulating mitophagy and promotes amniotic fluid stem cells to repair diabetic nephropathy	[38] [39]
OGT (O-linked N-acetylglucosamine (O-GlcNAc) transferase)	OGT ensures mitochondrial quality through mitophagy, thereby regulating the maintenance and stress response of hematopoietic stem cells	[40]
NOD2 (domain-containing protein 2)	NOD2 mediates the protection of LGR5 (Leucine-rich repeat-containing G-protein coupled receptor 5) intestinal stem cells against ROS cytotoxicity through mitophagy stimulation	[41]
TGF-β (Transforming growth factor β)	TGF-β1 enhances and accelerates the in vitro red blood cell formation of hematopoietic stem cells via stimulating mitophagy TGF-β involves in the differentiation of chicken embryonic stem cells into male germ cells	[42] [43]
HSPA1L (heat shock 70 kDa protein 1L)	Melatonin inhibits senescence-derived mitochondrial dysfunction in mesenchymal stem cells through the HSPA1L-mitophagy pathway	[44]
miRNA -322	MiRNA-322can self-renew and regulate mouse spermatogenic stem cells	[45]
p53	Down-regulation of p53 expression can reduce the accumulation of mitochondria in damaged cells and effectively resist stress-induced apoptosis and senescence of BMSCs Mitophagy controls the activity of p53 to regulate liver cancer stem cells	[46] [47]
FOXO3 (forkhead box O3)	FOXO3 is involved in the control of mitochondrial function of HSCs	[48]
2-hg (2-hydroxyglutaric acid)	Mitochondrial metabolism influences the fate of HSCs and determines their role in hematopoietic cells through 2-hg	[49]
Apaf-1 (apoptotic enzyme activation factor)	The lower expression of Apaf-1 in early differentiation of human embryonic neural stem cells against apoptosis	[50]
ISG15 (interferon-stimulated gene 15)	ISG15 is essential for optimized and effective OXPHOS, as it ensures the circulation of dysfunctional mitochondria, and when absent, a dysregulation in mitophagy occurs that negatively impacts pancreatic cancer stem cells (PaCSCs) stemness	[51]
MF (Mangiferin)	MF promotes the phenotype of brown fat cells by inhibiting the mitophagy of mesenchymal stem cells	[52]
BAG5 (BCL2 associated athanogene 5)	Decrease of BAG5 leads to the instability of PINK1, thereby damaging mitophagy	[53]
mir-351-5p	The mitochondrial fission and accompanying mitophagy by miR-351-5p/Miro2 axis is critical in hippocampal neural progenitor cell death, and a potential therapeutic target in AD	[54]
Moringin	Moringin inhibits the expression of genes involved in mitophagy in human periodontal ligament stem cells	[55]
C89 (the small-molecule compound 89)	C89 induced autophagy involves in development and death of female germ stem cells through PI3K-AKT pathway	[56]
Memantine	Memantine enhances the mitochondrial degradation induced by iPSCs, and accelerated the clearance of damaged mitochondria through PINK1/parkin-mediated mitophagy	[57]
Pioglitazone	Pioglitazone has a significant inhibitory effect on autophagy of bone marrow mesenchymal stem cells, and it can protect mesenchymal stem cells from p-methylphenol-induced mitochondrial dysfunction by upregulation of PINK1	[58]
Printex 90	Printex 90 can inhibit the osteogenic differentiation and mitochondrial dysfunction of MSCs, and affect the regulation of mitochondrial biogenesis, kinetics and mitosis	[59]
Doxycycline (DOX)	The potent inhibition of EMT (epithelial-to-mesenchymal transition) and cancer stem-like characteristics in breast cancer cells by DOX treatment	[60]
PTBP1 (polypyrimidine binding protein 1)	Treatment with PTBP1 transformed the mitochondrial metabolism of CSCs in the colon to aerobic glycolysis, which may be related to the change in the characteristics of CSCs in the colon	[61]
Atad3a (ATPase family AAA-domain containing protein 3A)	Deletion of Atad3a induces hyperactivated mitophagy through Parkin/Pink1 pathway and impairs the homeostasis of HSCs and progenitor cells	[62]
DXR (doxorubicin)	The mitophagy level and expression of BNIP3L, a mitophagy regulator, were significantly higher in CSCs than in parental cells after DXR treatment	[63]
miR-1	Overexpression of a miR-1 could destroy mitochondria of cancer stem cells and induced mitophagy of cancer stem cells	[64]
Salinomycin	Salinomycin can induce mitophagy in some cells and may promote the differentiation of tumor stem cells by targeting the Wnt/β-catenin signaling pathway, thus eliminating tumor stem cells	[65]
DCA (dichloroacetate)	DCA can affect stemness-associated characteristics and mitochondrial function of pancreatic cancer cell lines	[66]
Nrf2 (nuclear factor erythroid 2-related factor 2)	Nrf2 is an essential molecule in the maintenance of CSCs’ stemness and self-renewal in response to different oxidative stresses such as chemotherapy-induced elevation of ROS	[67]

A reduction in mitophagy can induce a variety of diseases and cause different cancers through various factors. The extent to which mitophagy promotes stem cell “stemness” is not fully understood andis dependent on the stem cell type. Therefore, the mechanism of mitophagy still needs to be further explored, suggesting that we should continue to study and provide new insights for treating mitophagy-related diseases in the future. Stem cells are the “seed cells” for various tissues and organs, and exploring the role and mechanism of mitochondria in stem cells, especially in differentiation, self-renewal and apoptosis, will providenovel insights into regenerative medicine and cancer treatment. Understanding how to improve the role of mitophagy in stem cells to improve clinical efficacy, including the elimination of residual disease, is important. Taken together, the role of mitophagy and underlying mechanism in stem cells need to be further explored.

## Supplementary Information

**Additional file 1.** The references of tables.

## Data Availability

Not applicable.

## Abbreviations

Baf: Bafilomycin A1
BNIP3: BCL2 interacting protein 3
BNIP3L/NIX: BCL2/adenovirus E1B interacting protein 3-like
DNM1L: Dynamin 1-like
FUNDC1: FUN14 domain containing 1
LC3B: Microtubule-associated protein 1 light chain 3
MFN1/2: Mitofusin 1/2
PHB2: Prohibitin 2
SQSTM1: Sequestosome 1
PRDX6: Peroxiredoxin 6
PINK1: PTEN-induced putative kinase 1
PRKN/Parkin: Parkin RBR E3 ubiquitin protein ligase
ROS: Reactive oxygen species
NOD2: Binding oligomerization domain-containing protein 2
MDP: Muramyl dipeptide
TIM23: Translocase of the inner mitochondrial membrane
VDAC1: Voltage-dependent anion channel 1
FIS1: Mitochondrial fission protein 1
Atad3a: ATPase family, AAA domain containing 3A
AD: Alzheimer’s disease
ONFH: Osteonecrosis of the femoral head
AKT: Protein kinase B
HSPA1L: Heat shock 70 kDa protein 1L
ADMSCs: Adipose-derived mesenchymal stem cells
IR: Ischemia reperfusion
Atg16L1: Autophagy related protein 16 like protein 1
AFSC: Amniotic fluid stem cell
AMPK: AMP-activated protein kinase
SIRT3: Sirtuin (silent mating type information regulation 2 homolog) 3
