# Supplementary material for: New insights into mitophagy and stem cells
Source: Stem Cell Res Ther. 2021 Aug 11;12:452. doi: 10.1186/s13287-021-02520-5 (PMC8359610; doi:10.1186/s13287-021-02520-5)
Supplement: Supplementary file 1 — Additional file 1. The references of tables. [file 13287_2021_2520_MOESM1_ESM.docx]

1 Wiseman S, et al. Expression of gp78/autocrine motility factor receptor and endocytosis of autocrine motility factor in human thyroid cancer cells. Cureus 2019; 11: e4928 [PMID:31431834 DOI: 10.7759/cureus.4928]

2 Xiang L, et al. Hypoxia-inducible factor 1 mediates taz expression and nuclear localization to induce the breast cancer stem cell phenotype. Oncotarget 2014; 5: 12509-12527 [PMID:25587023 DOI: 10.18632/oncotarget.2997]

3 He R, et al. Hif1a alleviates compression-induced apoptosis of nucleus pulposus derived stem cells via upregulating autophagy. Autophagy 2021, 10.1080/15548627.2021.1872227: 1-23 [PMID:33455530 DOI: 10.1080/15548627.2021.1872227]

4 Onorati M, et al. Zika virus disrupts phospho-tbk1 localization and mitosis in human neuroepithelial stem cells and radial glia. Cell reports 2016; 16: 2576-2592 [PMID:27568284 DOI: 10.1016/j.celrep.2016.08.038]

5 Catanese A, et al. Retinoic acid worsens atg10-dependent autophagy impairment in tbk1-mutant hipsc-derived motoneurons through sqstm1/p62 accumulation. Autophagy 2019; 15: 1719-1737 [PMID:30939964 DOI: 10.1080/15548627.2019.1589257]

6 Tucker B, et al. Duplication of tbk1 stimulates autophagy in ipsc-derived retinal cells from a patient with normal tension glaucoma. Journal of stem cell research & therapy 2014; 3: 161 [PMID:24883232 DOI: 10.4172/2157-7633.1000161]

7 Kim J, et al. Tbk1 regulates prostate cancer dormancy through mtor inhibition. Neoplasia (New York, NY) 2013; 15: 1064-1074 [PMID:24027431 DOI: 10.1593/neo.13402]

8 Wang C, et al. Pink1-mediated mitophagy maintains pluripotency through optineurin. Cell proliferation 2021; 54: e13034 [PMID:33931895 DOI: 10.1111/cpr.13034]

9 Liu Z, et al. Autophagy receptor optn (optineurin) regulates mesenchymal stem cell fate and bone-fat balance during aging by clearing fabp3. Autophagy 2020, 1-17 [PMID: 33143524 DOI: 10.1080/15548627.2020.1839286]

10 Jung J, et al. Mitochondrial nix promotes tumor survival in the hypoxic niche of glioblastoma. Cancer research 2019; 79: 5218-5232[PMID:31488423DOI: 10.1158/0008-5472.Can-19-0198]

11 Lee H, et al. Bnip3 induction by hypoxia stimulates fasn-dependent free fatty acid production enhancing therapeutic potential of umbilical cord blood-derived human mesenchymal stem cells. Redox biology 2017; 13: 426-443 [PMID:28704726 DOI: 10.1016/j.redox.2017.07.004]

12 Xue Y, et al. Angelica polysaccharide moderates hypoxia-evoked apoptosis and autophagy in rat neural stem cells by downregulation of bnip3. Artificial cells, nanomedicine, and biotechnology 2019; 47: 2492-2499 [PMID:31208217 DOI: 10.1080/21691401.2019.1623228]

13 Li F, et al. Mesenchymal stem cell-derived extracellular vesicles prevent neural stem cell hypoxia injury via promoting mir-210-3p expression. Molecular medicine reports 2020; 22: 3813-3821 [PMID: 33000190 DOI: 10.3892/mmr.2020.11454]

14 Zhang J, et al. Endothelial monocyte-activating polypeptide-ii induces bnip3-mediated mitophagy to enhance temozolomide cytotoxicity of glioma stem cells via down-regulating mir-24-3p. Frontiers in molecular neuroscience 2018; 11: 92 [PMID:29632473 DOI: 10.3389/fnmol.2018.00092]

15 Li E, et al. Bmal1 regulates mitochondrial fission and mitophagy through mitochondrial protein bnip3 and is critical in the development of dilated cardiomyopathy. Protein & cell 2020; 11: 661-679 [PMID:32277346 DOI: 10.1007/s13238-020-00713-x]

16 Koehler C, et al. Drosophilapink1 and parkin regulate intestinal stem cell proliferation during stress and aging. The Journal of cell biology 2017; 216: 2315-2327 [PMID:28663346 DOI: 10.1083/jcb.201610036]

17 Park H, et al. Parkin promotes mitophagic cell death in adult hippocampal neural stem cells following insulin withdrawal. Frontiers in molecular neuroscience 2019; 12: 46 [PMID:30853892 DOI: 10.3389/fnmol.2019.00046]

18 Esteca M, et al. Loss of parkin results in altered muscle stem cell differentiation during regeneration. International journal of molecular sciences 2020; 21: [PMID:33126429 DOI: 10.3390/ijms21218007]

19 Rakovic A, et al. Phosphatase and tensin homolog (pten)-induced putative kinase 1 (pink1)-dependent ubiquitination of endogenous parkin attenuates mitophagy: Study in human primary fibroblasts and induced pluripotent stem cell-derived neurons. The Journal of biological chemistry 2013; 288: 2223-2237 [PMID: 23212910 DOI: 10.1074/jbc.M112.391680]

20 Lv Y, et al. Melatonin attenuates chromium (vi)-induced spermatogonial stem cell/progenitor mitophagy by restoration of mettl3-mediated rna n-methyladenosine modification. Frontiers in cell and developmental biology 2021; 9: 684398 [PMID:34150779 DOI: 10.3389/fcell.2021.684398]

21 Hsieh C, et al. Functional impairment in miro degradation and mitophagy is a shared feature in familial and sporadic parkinson's disease. Cell stem cell 2016; 19: 709-724 [PMID:27618216 DOI: 10.1016/j.stem.2016.08.002]

22 Ahmad T, et al. Miro1 regulates intercellular mitochondrial transport & enhances mesenchymal stem cell rescue efficacy. The EMBO journal 2014; 33: 994-1010 [PMID:24431222 DOI: 10.1002/embj.201386030]

23 Pei S, et al. Ampk/fis1-mediated mitophagy is required for self-renewal of human aml stem cells. Cell stem cell 2018; 23: 86-100.e106 [PMID:29910151 DOI: 10.1016/j.stem.2018.05.021]

24 Liu D, et al. The mitochondrial fission factor fis1 promotes stemness of human lung cancer stem cells via mitophagy. FEBS open bio 2021; 11: 1997-2007 [PMID:34051059 DOI: 10.1002/2211-5463.13207]

25 Wang L, et al. Drp1 is dispensable for mitochondria biogenesis in induction to pluripotency but required for differentiation of embryonic stem cells. Stem cells and development 2014; 23: 2422-2434 [PMID:24937776 DOI: 10.1089/scd.2014.0059]

26 Gong J, et al. Phosphorylation of ulk1 by ampk is essential for mouse embryonic stem cell self-renewal and pluripotency. Cell death & disease 2018; 9: 38 [PMID:29348566 DOI: 10.1038/s41419-017-0054-z]

27 Vorobev M, et al. The upregulation of ulk1-dependent autophagy does not require the p53 activity in mouse embryonic stem cells. Biochemical and biophysical research communications 2021; 552: 78-83 [PMID:33743351 DOI: 10.1016/j.bbrc.2021.03.034]

28 Li Z, et al. Mitochondrial phosphoenolpyruvate carboxykinase regulates osteogenic differentiation by modulating ampk/ulk1-dependent autophagy. Stem cells (Dayton, Ohio) 2019; 37: 1542-1555 [PMID:31574189 DOI: 10.1002/stem.3091]

29 Kowno M, et al. Prohibitin 2 regulates the proliferation and lineage-specific differentiation of mouse embryonic stem cells in mitochondria. PloS one 2014; 9: e81552 [PMID:24709813DOI: 10.1371/journal.pone.0081552]

30 Chen L, et al. Apelin-13 induces mitophagy in bone marrow mesenchymal stem cells to suppress intracellular oxidative stress and ameliorate osteoporosis by activation of ampk signaling pathway. Free radical biology & medicine 2021; 163: 356-368 [PMID:33385540 DOI: 10.1016/j.freeradbiomed.2020.12.235]

31 Yin F, et al. Bone marrow mesenchymal stem cells repair cr (vi)- injured kidney by regulating mitochondria-mediated apoptosis and mitophagy mediated via the mapk signaling pathway. Ecotoxicology and environmental safety 2019; 176: 234-241 [PMID:30939403 DOI: 10.1016/j.ecoenv.2019.03.093]

32 Maity J, et al. Klf2 regulates dental pulp-derived stem cell differentiation through the induction of mitophagy and altering mitochondrial metabolism. Redox biology 2020; 36: 101622 [PMID:32777717 DOI: 10.1016/j.redox.2020.101622]

33 Kim J, et al. Pedf-mediated mitophagy triggers the visual cycle by enhancing mitochondrial functions in a ho-injured rat model. Cells 2021; 10: [PMID:34066394 DOI: 10.3390/cells10051117]

34 Zhao L, et al. Transbhlhe40/sirt1 axis-regulated mitophagy is implicated in all- retinoic acid-induced spina bifida aperta. Frontiers in cell and developmental biology 2021; 9: 644346 [PMID:33987177 DOI: 10.3389/fcell.2021.644346]

35 Sun J, et al. Targeting histone deacetylase sirt1 selectively eradicates egfr tki-resistant cancer stem cells via regulation of mitochondrial oxidative phosphorylation in lung adenocarcinoma. Neoplasia (New York, NY) 2020; 22: 33-46 [PMID:31765940 DOI: 10.1016/j.neo.2019.10.006]

36 Poon A, et al. The role of sqstm1 (p62) in mitochondrial function and clearance in human cortical neurons. Stem cell reports 2021; 16: 1276-1289 [PMID:33891871 DOI: 10.1016/j.stemcr.2021.03.030]

37 Liu F, et al. Lrrc17 controls bmsc senescence via mitophagy and inhibits the therapeutic effect of bmscs on ovariectomy-induced bone loss. Redox biology 2021; 43: 101963 [PMID:33865167 DOI: 10.1016/j.redox.2021.101963]

38 Guo Y, et al. Sirt3-mediated mitophagy regulates ages-induced bmscs senescence and senile osteoporosis. Redox biology 2021; 41: 101915 [PMID:33662874 DOI: 10.1016/j.redox.2021.101915]

39 Feng J, et al. Sirt3 facilitates amniotic fluid stem cells to repair diabetic nephropathy through protecting mitochondrial homeostasis by modulation of mitophagy. Cellular physiology and biochemistry : international journal of experimental cellular physiology, biochemistry, and pharmacology 2018; 46: 1508-1524 [PMID:29689547 DOI: 10.1159/000489194]

40 Murakami K, et al. Ogt regulates hematopoietic stem cell maintenance via pink1-dependent mitophagy. Cell reports 2021; 34: 108579 [PMID:33406421 DOI: 10.1016/j.celrep.2020.108579]

41 Levy A, et al. Innate immune receptor nod2 mediates lgr5 intestinal stem cell protection against ros cytotoxicity via mitophagy stimulation. Proceedings of the National Academy of Sciences of the United States of America 2020; 117: 1994-2003 [PMID:31919280 DOI: 10.1073/pnas.1902788117]

42 Kuhikar R, et al. Transforming growth factor β1 accelerates and enhances in vitro red blood cell formation from hematopoietic stem cells by stimulating mitophagy. Stem cell research &therapy 2020; 11: 71 [PMID:32075694 DOI: 10.1186/s13287-020-01603-z]

43 Zhang Y, et al. Effects of the transforming growth factor beta signaling pathway on the differentiation of chicken embryonic stem cells into male germ cells. Cellular reprogramming 2016; 18: 401-410 [PMID:27906584 DOI: 10.1089/cell.2016.0019]

44 Lee J, et al. Melatonin suppresses senescence-derived mitochondrial dysfunction in mesenchymal stem cells via the hspa1l-mitophagy pathway. Aging cell 2020; 19: e13111 [PMID:31965731 DOI: 10.1111/acel.13111]

45 Wang Y, et al. Rassf8microrna-322 regulates self-renewal of mouse spermatogonial stem cells through. International journal of biological sciences 2019; 15: 857-869 [PMID:30906216 DOI: 10.7150/ijbs.30611]

46 Zhang F, et al. P53 and parkin co-regulate mitophagy in bone marrow mesenchymal stem cells to promote the repair of early steroid-induced osteonecrosis of the femoral head. Cell death & disease 2020; 11: 42 [PMID:31959744 DOI: 10.1038/s41419-020-2238-1]

47 Liu K, et al. Mitophagy controls the activities of tumor suppressor p53 to regulate hepatic cancer stem cells. Molecular cell 2017; 68: 281-292.e285 [PMID:29033320 DOI: 10.1016/j.molcel.2017.09.022]

48 Liang R, Ghaffari S. Mitochondria and foxo3 in stem cell homeostasis, a window into hematopoietic stem cell fate determination. Journal of bioenergetics and biomembranes 2017; 49: 343-346 [PMID:28639090 DOI: 10.1007/s10863-017-9719-7]

49 Ansó E, et al. The mitochondrial respiratory chain is essential for haematopoietic stem cell function. Nature cell biology 2017; 19: 614-625 [PMID:28504706 DOI: 10.1038/ncb3529]

50 Karimzadeh S, et al. Insufficient apaf-1 expression in early stages of neural differentiation of human embryonic stem cells might protect them from apoptosis. European journal of cell biology 2018; 97: 126-135 [PMID:29395479 DOI: 10.1016/j.ejcb.2018.01.005]

51 Alcalá S, et al. Isg15 and isgylation is required for pancreatic cancer stem cell mitophagy and metabolic plasticity. Nature communications 2020; 11: 2682 [PMID:32472071 DOI: 10.1038/s41467-020-16395-2]

52 Whitworth AJ, Pallanck LJ. Pink1/parkin mitophagy and neurodegeneration-what do we really know in vivo? Curr Opin Genet Dev 2017; 44: 47-53 [PMID:28213158 DOI: 10.1016/j.gde.2017.01.016]

53 Tsujimoto T, et al. Mir-155 inhibits mitophagy through suppression of bag5, a partner protein of pink1. Biochemical and biophysical research communications 2020; 523: 707-712 [PMID:31948758 DOI: 10.1016/j.bbrc.2020.01.022]

54 Woo H, et al. Mir-351-5p/miro2 axis contributes to hippocampal neural progenitor cell death via unbalanced mitochondrial fission. Molecular therapy Nucleic acids 2021; 23: 643-656 [PMID:33575111 DOI: 10.1016/j.omtn.2020.12.014]

55 Chiricosta L, et al. Moringin pretreatment inhibits the expression of genes involved in mitophagy in the stem cell of the human periodontal ligament. Molecules (Basel, Switzerland) 2019; 24: [PMID:31487916 DOI: 10.3390/molecules24183217]

56 Li X, et al. C89 induces autophagy of female germline stem cells via inhibition of the pi3k-akt pathway in vitro. Cells 2019; 8: [PMID:31216656 DOI: 10.3390/cells8060606]

57 Hirano K, et al. Neuroprotective effects of memantine via enhancement of autophagy. Biochemical and biophysical research communications 2019; 518: 161-170 [PMID:31431260 DOI: 10.1016/j.bbrc.2019.08.025]

58 Yoon Y, et al. Ppioglitazone protects mesenchymal stem cells against -cresol-induced mitochondrial dysfunction via up-regulation of pink-1. International journal of molecular sciences 2018; 19: [PMID:30250007 DOI: 10.3390/ijms19102898]

59 Shen Y, et al. Carbon black suppresses the osteogenesis of mesenchymal stem cells: The role of mitochondria. Particle and fibre toxicology 2018; 15: 16 [PMID:29650039 DOI: 10.1186/s12989-018-0253-5]

60 Zhang L, et al. Doxycycline inhibits the cancer stem cell phenotype and epithelial-to-mesenchymal transition in breast cancer. Cell cycle (Georgetown, Tex) 2017; 16: 737-745 [PMID:27753527 DOI: 10.1080/15384101.2016.1241929]

61 Choi J, et al. Metabolic influence of walnut phenolic extract on mitochondria in a colon cancer stem cell model. European journal of nutrition 2019; 58: 1635-1645 [PMID:29740695 DOI: 10.1007/s00394-018-1708-z]

62 Yang C, Suda TJNi. Hyperactivated mitophagy in hematopoietic stem cells. 2018; 19: 2-3 [PMID:29242545 DOI: 10.1038/s41590-017-0008-8]

63 Yan C, et al. Doxorubicin-induced mitophagy contributes to drug resistance in cancer stem cells from hct8 human colorectal cancer cells. Cancer letters 2017; 388: 34-42 [PMID:27913197 DOI: 10.1016/j.canlet.2016.11.018]

64 Zhang S, et al. Mitochondrial damage mediated by mir-1 overexpression in cancer stem cells. Molecular therapy Nucleic acids 2019; 18: 938-953 [PMID:31765945 DOI: 10.1016/j.omtn.2019.10.016]

65 Jangamreddy J, et al. Salinomycin induces activation of autophagy, mitophagy and affects mitochondrial polarity: Differences between primary and cancer cells. Biochimica et biophysica acta 2013; 1833: 2057-2069 [PMID:23639289 DOI: 10.1016/j.bbamcr.2013.04.011]

66 Tataranni T, et al. Dichloroacetate affects mitochondrial function and stemness-associated properties in pancreatic cancer cell lines. Cells 2019; 8: [PMID:31109089 DOI: 10.3390/cells8050478]

67 Payandeh Z, et al. Role of nrf2 and mitochondria in cancer stem cells; in carcinogenesis, tumor progression, and chemoresistance. Biochimie 2020; 179: 32-45 [PMID:32946993 DOI: 10.1016/j.biochi.2020.09.014]
